# Islamic microfinance institution: Survey data from Indonesia

**DOI:** 10.1016/j.dib.2019.104911

**Published:** 2019-11-30

**Authors:** Bayu Arie Fianto

**Affiliations:** Department of Shari'a Economics, Faculty of Economics and Business, Universitas Airlangga, Surabaya, Indonesia

**Keywords:** Profit and loss sharing, Islamic microfinance institutions, Financing, Indonesia

## Abstract

This article focuses on presenting a two-year panel data set of Islamic microfinance institutions' (MFIs) clients in Indonesia. The clients are divided into three groups: clients receiving profit and loss sharing (PLS) finance, receiving non-PLS finance and those receiving finance by both mechanisms. Data obtained from 289 Indonesian Islamic MFIs clients contain the clients' financing characteristics. The chi-square (χ^2^) inferential statistics model was used in the data analysis. The data show that total finance approved, the duration of the financing application process, the financing duration, and savings in Islamic MFIs is associated with the three groups.

**Table:** Specifications Table

Subject	Economics and Finance
Specific subject area	Finance
Type of data	Table
How data were acquired	Field survey, face to face interview
Data format	Raw
Parameters for data collection	Sample comprises clients receiving finance from Islamic MFIs
Description of data collection	Two-year panel data set of Islamic MFIs' clients. Data were obtained through questionnaires administered to rural households that received finance from Islamic MFIs in East Java, Indonesia.
Data source location	Three regions (Kediri, Tulungagung, and Pasuruan) in East Java, Indonesia
Data accessibility	Data included in this article
Related research article	B.A. Fianto, C. Gan, B. Hu, and J. Roudaki, Equity financing and debt based financing: Evidence from Islamic microfinance institutions in Indonesia, Pacific-Basin Finance Journal. https://doi.org/10.1016/j.pacfin.2017.09.010 [1].

**Table:** 

**Value of the Data** • These data describe the financial characteristics of Islamic MFIs' clients in Indonesia. These data are useful for researchers who investigate Islamic MFIs especially their financing mechanisms. These data are also important for regulators. Most governments aim to reduce poverty and assist the poor through microfinancing. These data may help give the detailed characteristics of clients relative to their financing mechanism. • The data show that Islamic MFI clients have different financing mechanisms. In general, clients are in three groups: clients receiving PLS funding mechanisms, clients receiving non-PLS funding mechanisms, and clients receiving by both financing mechanisms. The three groups show slightly different characteristics. • These data might be used for further research especially on measuring impact of financing or access to financing for the three client groups that received finance by the different financing mechanisms.

## 1 Data description

This data used a structured survey questionnaire to obtain data on the financing characteristics and government assistance for Islamic MFIs' clients. There were 289 clients from four Islamic MFIs in East Java, Indonesia. A random pilot test with a sample of 15 clients in East Java was conducted before administering the survey. Data were analysed using Tables, percentages and chi-square (χ^2^) test at 5% and 1% level of significance.

## 2 Experimental design, materials, and methods

Table 1 provides general information about Islamic MFIs' clients and their financing characteristics. Based on the survey, there are three groups of clients. First are clients who received finance by a profit and loss sharing (PLS) financing mechanism, followed by clients who received finance by a non-PLS mechanism and, thirdly, clients who received finance by both PLS and non-PLS mechanisms. The total number of clients was 289, consisting of 112 clients with a PLS mechanism, 162 clients with a non-PLS mechanism, and 15 clients with both mechanisms. Most non-PLS clients received IDR 1 million to 3 million,^1^ whereas most PLS clients received 3 million to 5 million. The PLS group had a higher amount of finance because they are more trusted by Islamic MFIs. The chi-square test for differences in the amount of finance received by clients is significant at the 5% level which implies that the amount of finance received by clients is influenced by the type of financing mechanism.

**Table 1:** Financing characteristics of Islamic MFIs' clients.

	Clients with PLS (N_1_ = 112)		Clients with non-PLS (N_2_ = 162)		Mixed PLS and non-PLS (N_3_ = 15)		All Clients (N = 289)		Statistical Test
	Count (n_1_)	% of N_1_	Count (n_2_)	% of N_2_	Count (n_3_)	% of N_3_	Count (n = n_1_+n_2_+n_3_)	% of N	
**Total amount of finance approved**									*χ*^2^ = 22.019^a^
Less than IDR 1,000,000	6	5.5	6	3.8	0	0.0	12	4.3	
Between IDR 1,000,001 and 3,000,000	28	25.7	42	26.9	2	13.3	72	25.7	
Between IDR 3,000,001 and 5,000,000	32	29.4	35	22.4	1	6.7	68	24.3	
Between IDR 5,000,001 and 7,000,000	18	16.5	14	9.0	1	6.7	33	11.8	
Between IDR 7,000,001 and 15,000,000	15	13.8	26	16.7	6	40.0	47	16.8	
More than IDR 15,000,000	10	9.2	33	21.2	5	33.3	48	17.1	
Sub Total	109	100.0	156	100.0	15	100.0	280	100.0	
**Time to process**									*χ*^2^ = 24.975^b^
< than a week	102	91.1	123	76.9	9	60.0	234	81.5	
1 week	5	4.5	17	10.6	5	33.3	27	9.4	
2 weeks	2	1.8	17	10.6	0	0.0	19	6.6	
3 weeks	3	2.7	3	1.9	1	6.7	7	2.4	
1 Month	0	0.0	0	0.0	0	0.0	0	0.0	
> a month	0	0.0	0	0.0	0	0.0	0	0.0	
Sub Total	112	100.0	160	100.0	15	100.0	287	100.0	
**Duration of financing**									*χ*^2^ = 21.142^b^
3–6 months	53	53.5	43	29.5	3	23.1	99	38.4	
7–12 months	26	26.3	57	39.0	5	38.5	88	34.1	
1–2 years	18	18.2	33	22.6	5	38.5	56	21.7	
2–3 years	2	2.0	8	5.5	0	0.0	10	3.9	
> than 3 years	0	0.0	5	3.4	0	0.0	5	1.9	
Sub Total	99	100.0	146	100.0	13	100.0	258	100.0	
**Required collateral**									*χ*^2^ = 4.100
No	1	0.9	8	4.9	0	0.0	9	3.1	
Yes	111	99.1	154	95.1	15	100.0	280	96.9	
Sub Total		100.0		100.0		100.0		100.0	
**Having savings in Islamic MFI**									*χ*^2^ = 11.351^b^
No	32	28.8	25	15.4	0	0.0	57	19.8	
Yes	79	71.2	137	84.6	15	100.0	231	80.2	
Sub Total	111	100.0	162	100.0	15	100.0	288	100.0	

a 5% significance level.

b 1% significance level.

Table 1 also compares the processing time to obtain finance among clients with the PLS, non-PLS, and mixed mechanisms. The data show that 91.1% of clients with PLS mechanisms obtained their finance from an Islamic MFI in less than a week after submitting their application compared with 76.9% of clients with non-PLS mechanisms and 60% for clients with mixed mechanisms. This shows that, on average, Islamic MFIs take only a short time to process the clients' loan applications. There is a statistically significant difference (at the 1% level) for this variable between the three groups. This implies that the finance application process time is associated with the financing type.

Regarding financing duration, for most clients with a PLS mechanism it was between three and six months (53.5%). For clients with a non-PLS mechanism, it was between seven and 12 months (39%) and for clients in both mechanisms, between seven and 12 months (38.5%) and between one and two years (38.5%). These data are statistically significant at the 1% level between the three groups. This implies that financing duration contributes significantly to the clients' financing type. In terms of payment, most respondents chose monthly as their mode of payment (over 85%). Most clients in the three groups answered that their financing requires collateral (over 95%). For clients with PLS mechanisms, only 0.9% did not provide collateral; it was 4.9% for clients with non-PLS mechanisms and for clients with mixed mechanisms all provided collateral for their finance. In terms of saving with an Islamic MFI, about 71.2% of the clients in the PLS mechanisms had been saving with Islamic MFIs. This is slightly lower than clients with non-PLS mechanisms (84.6%). All clients in the both mechanisms group had been saving with an Islamic MFI. The differences are statistically significant at the 1% level. This means that saving with an Islamic MFI influences the type of finance mechanism from Islamic MFIs.

This data also investigated the Indonesian government's support for Islamic MFI clients during their financing period. The data show that only 34 (11.8%) of clients received assistance and support from the government. This comprises 20% of clients with both funding mechanisms, followed by clients 12.4% with non-PLS mechanisms and 9.8% with PLS mechanisms. Most government assistance was financial (52.6%), followed by skill/technical support (21.1%); only 10.5% of clients received religious support (see Table 2).

**Table 2:** Government assistance to Islamic MFIs' clients.

	Clients with PLS (N_1_ = 112)		Clients with non-PLS (N_2_ = 162)		Mixed PLS and non-PLS (N_3_ = 15)		All Clients (N = 289)		Statistical Test
	Count (n_1_)	% of n_1_	Count (n_2_)	% of n_2_	Count (n_3_)	% of n_3_	Count (n = n_1_+n_2_+n_3_)	% of n	
**Assistance from the govt. after finance**									*χ*^2^ = 1.450
No	101	90.2	141	87.6	12	80.0	254	88.2	
Yes	11	9.8	20	12.4	3	20.0	34	11.8	
Sub Total	112	100.0	161	100.0	15	100.0	288	100.0	
**Frequency of assistance**									
Once	9	81.8	13	65.0	1	33.3	23	67.6	*χ*^2^ = 4.402
Twice	2	18.2	5	25.0	2	66.7	9	26.5	
Three times	0	0.0	0	0.0	0	0.0	0	0.0	
Over three times	0	0.0	2	10.0	0	0.0	2	5.9	
Sub Total	11	100.0	20	100.0	3	100.0	34	100.0	
**Assistance beneficial**									
No	1	9.1	0	0.0	0	0.0	1	2.9	*χ*^2^ = 2.154
Yes	10	90.9	20	100.0.0	3	100.0.0	33	97.1	
Sub Total	11	100.0	20	100.0	3	100.0	34	100.0	

With regard to the frequency of government assistance, over two thirds of clients with both mechanisms had received help twice (66.7%) followed by non-PLS clients (25.0%) and PLS clients (18.2%). A high proportion (81.8%) of PLS clients and nearly two thirds (65.0%) of non-PLS clients received help only once. When respondents were asked whether the assistance benefitted them, most (97.1%) replied that the support had helped their household. They also believed that once they received assistance or support from the government, it meant they had a better chance of getting future assistance (32.6%). Another benefit was that some clients felt that the support increased their business knowledge (30.2%).

## Footnotes

1 In November 2019, 1 USD equaled 14,076 IDR (Indonesian rupiah).

## References

[1] Fianto B.A., Gan C., Hu B., Roudaki J. (2018). Equity financing and debt-based financing: evidence from Islamic microfinance institutions in Indonesia. Pac. Basin Financ. J..

