# Supplementary material for: Islamic microfinance institution: Survey data from Indonesia
Source: Data Brief. 2019 Nov 30;28:104911. doi: 10.1016/j.dib.2019.104911 (PMC6909144; doi:10.1016/j.dib.2019.104911)
Supplement: Multimedia component 1 [file mmc1.doc]

**Islamic Microfinance Institutions Financing’ Survey**

*Instructions: For each question with brackets provided, please tick your answer(s); otherwise, please follow the instructions given to answer the questions.* *Your participation is voluntary, and your answers will be kept confidential.*

| Section 1. Clients of Islamic MFIs |
| --- |

1. Did you borrow from any Islamic MFI over the last 2 years?

a. YES [ ] b. NO [ ]

1. What is the type of your Islamic MFI financing?

a. Profit and Loss Sharing (PLS) [ ]

b. Non-Profit and Loss Sharing (Non-PLS) [ ]

c. Mixed PLS and non-PLS [ ]

3. What was the total amount of financing you applied from Islamic MFI?

a. Less than Rp. 1,000,000 [ ]

b. Between Rp. 1,000,001 and Rp. 3,000,000 [ ]

c. Between Rp. 3,000,001 and Rp. 5,000,000 [ ]

d. Between Rp. 5,000,001 and Rp. 7,000,000 [ ]

e. Between Rp. 7,000,001 and Rp. 15,000,000 [ ]

f. More than Rp. 15,000,000 [ ]

4. What was the total amount of financing approved by the Islamic MFI in your application?

a. Less than Rp. 1,000,000 [ ]

b. Between Rp. 1,000,001 and Rp. 3,000,000 [ ]

c. Between Rp. 3,000,001 and Rp. 5,000,000 [ ]

d. Between Rp. 5,000,001 and Rp. 7,000,000 [ ]

e. Between Rp. 7,000,001 and Rp. 15,000,000 [ ]

f. More than Rp. 15,000,000 [ ]

1. How long did the Islamic MFI take to process your financing application?

a. Less than a week [ ]

b. 1 week [ ]

c. 2 weeks [ ]

d. 3 weeks [ ]

e. 1 month [ ]

f. More than a month [ ]

6. What was the duration of your financing?

a. 3 to 6 months [ ]

b. 7 to 12 months [ ]

c. 1 to 2 years [ ]

d. 2 to 3 years [ ]

e. More than 3 years [ ]

7. Did your borrowing require collateral?

a. YES [ ] b. NO [ ]

8. Do you have savings with an Islamic MFI?

- 1. YES [ ] b. NO [ ]

| Section 2. Assistance and Support for Clients |
| --- |

1. Did you receive any assistance/support from the government after you obtained your financing?
2. YES [ ] b. NO [ ]

2. If YES How many times did you get assistance/support from the government during your financing period?

a. Once [ ]

b. Twice [ ]

c. Three times [ ]

d. More than three times [ ]

3. Did you find the assistance/support beneficial?

1. YES [ ] b. NO [ ]

*Your participation in this survey is greatly appreciated; thank you for your time. If you have further comments about your financing, please feel free to comment in the space provided below. Once again, we assure you that your identity will remain* ***STRICTLY CONFIDENTIAL.***
